# Somatostatin 5 receptor expression in prolactinomas: Is there a role for Pasireotide in the management of prolactinomas?

**DOI:** 10.1007/s11102-025-01580-7

**Published:** 2025-11-03

**Authors:** Nidhi Agrawal, Sonal Mehta, Richard A. Feelders, Samara Skwiersky, Claudia Campana, Fadime Dogan, Peter M. van Koetsveld, Sebastian J. C. M. M. Neggers, Kyla Wright, Hyon Kim, David Zagzag, Leo J. Hofland

**Affiliations:** 1 New York University Langone Medical Center, New York, NY USA; 2 Department of Internal Medicine, Division of Endocrinology, Erasmus MC Cancer Institute, Erasmus Medical Center, Rotterdam, The Netherlands; 3 NYU Langone Health - Long Island, Mineola, NY USA; 4 Endocrinology Unit, Department of Internal Medicine and Medical Specialties, School of Medical and Pharmaceutical Sciences, University of Genova, Genova, Italy; 5 Department of Surgery, University of California Los Angeles Health, Los Angeles, CA USA; 6 Robert Wood Johnson Barnabas Health, West Orange, NJ USA

**Keywords:** Somatostatin, Dopamine, Receptor, Prolactinoma, Resistance, Pasireotide, Octreotide, Cabergoline

## Abstract

The majority of prolactinomas are treated with dopamine agonists (DA) with excellent response, however DA-resistance occurs in 10% of prolactinomas. Somatostatin (SST) receptors have been increasingly studied in prolactinomas. There are five SST receptor subtypes and a significant number of prolactinomas show expression of SST_5_ and SST_1_ mRNA. The somatostatin analog (SSA) pasireotide, which has 40-fold greater binding affinity to SST_5_ compared to first-generation SSAs, shows promising results in case reports of DA-resistant prolactinomas. This two-center retrospective cohort study investigated the expression patterns of dopamine 2 (D2R), SST_2_ and SST_5_ receptors in surgical specimen of 34 patients with prolactinomas, 22 of which were DA-resistant. In vitro effects of cabergoline, octreotide and pasireotide on prolactin production was also examined in cultured prolactinoma cells. Receptor expression was scored using the immunoreactivity score (IRS). 31/34(91%) patients used DA preoperatively; 22/34(64.7%) were DA-resistant. Receptor expression in the cases was 97.1% for D2R, 70.6% for SST_5_ and 41.2% for SST_2_. In the majority of SST_2_ positive cases SST_2_ expression was very low. In in vitro studies comparing the effects of octreotide, pasireotide, and cabergoline on prolactin secretion, octreotide was the least potent drug and cabergoline was the most potent. SST_5_ and D2R expression was highest in prolactinomas showing the highest response to pasireotide and cabergoline in vitro (median D2R IRS 1.0 vs 8.0 for < 50% vs. > 50% inhibition by cabergoline and median SST_5_ IRS 3.5 avs. 12.0 for < 50% vs. > 50% inhibition by pasireotide). In a subgroup, pasireotide inhibited prolactin secretion with comparable potency to cabergoline. Targeting SST_5_ with pasireotide may be a potential treatment modality for further clinical investigation in the treatment of a subset of DA resistant or intolerant prolactinomas.

**Supplementary Information:**

The online version contains supplementary material available at 10.1007/s11102-025-01580-7.

## Introduction

Prolactinomas are the most common type of hormone-secreting pituitary tumor, accounting for 40% of all pituitary tumors [1]. The overall prevalence of prolactinomas in adults is estimated to be 60–100 per million [2]. Dopamine agonists (DAs), which act on dopamine D2 receptors (D2Rs), are typically highly expressed in prolactinomas. DAs can reduce prolactin (PRL) synthesis and secretion and induce tumor shrinkage [3].

Approximately 10% of prolactinomas are resistant to DAs, whereas up to 12% of patients are intolerant to DAs [4]. According to the most recent Pituitary Society international Consensus Statement, DA resistance is defined as a failure to achieve a normal PRL level or a $\:\ge\:30$% reduction in tumor size despite treatment with maximally tolerated doses of DA therapy for 3–6 months [5]. Response to dopamine agonist treatment may also be discrepant where tumor size is unchanged despite adequate biochemical response or vice versa. Failure to restore gonadal function including ovulation and fertility may also reflect treatment resistance [3].

Treatment options for DA-resistant prolactinomas include switching to another more potent DA, dose escalation of DA therapy beyond conventional doses, surgical resection, radiotherapy, and chemotherapy such as temozolomide [6].

The predominantly expressed dopamine receptor in prolactinomas is D2R and lower D2R expression is associated with DA resistance [7]. Prolactinomas also express somatostatin receptor (SST) subtypes, with SST_5_ and SST_1_ mRNA showing higher expression than SST_2_ [8, 9]. Pasireotide (PAS), a second-generation SRL, has a 40-fold greater affinity for SST_5_ than first-generation SRLs and may be a novel treatment option in patients intolerant or resistant to DA. Compared to OCT, PAS also binds with a higher affinity to SST_1_ and SST_3_, and has slightly lower binding affinity to SST_2_ [10, 11].

In order to assess whether PAS may be a potential therapeutic option in prolactinoma patients who are resistant to or intolerant of DA, we evaluated the tumoral SST_2_, SST_5_, and D2R expression in these patient groups and examined the in vitro effects of CAB, OCT, and PAS on prolactin secretion by cultured prolactinoma cells.

## Methods

### Patients

This is a two-center retrospective cohort study investigating the expression of D2R, SST_2_ and SST_5_ in surgical specimens of prolactinoma patients with DA-resistance, DA intolerance, or those who underwent surgery for other reasons (indicated below) (*n* = 34). The study included consecutive surgical cases of adult patients with a diagnosis of DA-resistant prolactinoma at NYU Langone Health and Erasmus MC from 01/01/1997 to 01/01/2022 (supplementary Table 1). We excluded patients with prolactinomas well-controlled on DA therapy, patients < 18 years old, pregnant patients, and patients with co-secreting pituitary tumors in order to keep the SST analysis specific to prolactinomas.

All medical data at Erasmus MC and NYU Langone Health were obtained retrospectively through patient charts. Prolactinoma specimens had been collected for diagnostic purposes and were de-identified before the analysis. The corresponding patient charts were reviewed, and data on age, sex, medical history, surgical history, radiographic findings, and histopathology of resected tumors were collected. Erasmus MC pathology data and samples were collected following their own legislation and methods. Permission from the Institutional Review Boards (IRBs) of Erasmus MC and NYU Langone Health were obtained. The study was performed retrospectively and according to the guidelines of the Central Committee on Research involving Human Subjects.

Through a Material Transfer Agreement, slides were shipped from NYU to Erasmus MC, Department of Internal Medicine, Division of Endocrinology, Rotterdam, The Netherlands.

### Immunohistochemical analysis of SST_2_, SST_5_ and D2R

Immunohistochemistry was performed on 4µm thick whole slide sections from formalin-fixed paraffin embedded (FFPE) tissue blocks, on a validated and accredited automated slide stainer (Benchmark ULTRA System, VENTANA Medical Systems, Tucson, AZ, USA) according to the manufacturer’s instructions. Briefly, following deparaffinization and heat-induced antigen retrieval, the tissue samples were incubated with the antibody of interest (SST_2_ rabbit monoclonal 1:25; Biotrend NB-49-015, SST_5_ rabbit monoclonal 1:200; Abcam ab109495 and D2R mouse monoclonal 1:800; Santa Cruz sc-5303) for 32 min at 37 °C, followed by Optiview detection (#760 − 700, Ventana). Counterstain was done by hematoxylin II for 12 min and a blue coloring reagent for 8 min. Each tissue slide contained a fragment of FFPE pancreas (SST_2_ and SST_5_) or normal pituitary gland (D2R) as an on-slide positive control.

Receptor expression was scored on the basis of the well-validated standardized immunoreactivity score (IRS). IRSs were calculated by multiplying integers representing the percentage of positively stained cells (> 80%, 4; 51–80%, 3; 10–50%, 2; <10%, 1; 0%, 0) and the staining intensity (strong, 3; moderate, 2; mild, 1; none, 0) per institutional protocol to produce a score from 0 (no staining) to 12 (maximum staining). Scores were calculated independently by two investigators [12]. In case of disagreement between scores, consensus was obtained by discussion.

### In vitro studies

The in vitro studies represent the retrospective analysis of data collected from 12 human prolactinoma cultures obtained between 2003 and 2020. In selected cases, both in vivo and in vitro data were available (supplementary Table 1). Single cell suspension of human prolactinoma tissue was obtained by enzymatic dispersion as described in detail previously. Cell culture methods and analysis of hormone secretion by cultured prolactinomas has been described in detail previously [13]. Data represent the results of 3-day incubations with OCT, PAS or CAB, unless indicated elsewhere. All drugs were tested at a concentration of 10nM. Part of the data on the effects of OCT and PAS (3 cases) have been previously described [13]. Due to the retrospective nature of the study and limitations with respect to the amount of tissue that was obtained, not all prolactinomas were tested simultaneously for all three drugs. The retrospective studies focused on PRL secretion only, not cell growth markers. In 10 cultures all three drugs were compared simultaneously. OCT and PAS (SOM230) were obtained from Novartis Pharma A.G. (Basel, Switzerland), CAB was obtained from Pfizer (New York, New York, US).

### Statistical analysis

Mean IRSs for SST_5_, SST_2_, and D2R were compared using unpaired t-tests between specimens from patients with and without the following clinico-radiological features: preoperative DA therapy, DA resistance, DA intolerance, apoplexy, cavernous sinus invasion, suprasellar extension, and optic chiasm compression. Correlation analysis between IRSs and continuous clinico-radiological features (i.e., tumor volume, preoperative PRL level) was performed. Analyses were completed in IBM SPSS (version 25, IBM Corp., Armonk, NY).

Comparison between the effects of OCT, PAS and CAB in vitro data was performed using one-way ANOVA. When significant overall effects were found, the Newman-Keuls multiple comparison test was used. To compare the effects of the drugs on PRL secretion with IRS scores, Mann Whitney test was used. Significance level was set to *p* < 0.05 for all analyses. For statistical analysis of in vitro data GraphPad Prism (version 9.0.0) was used.

## Results

### Patients

The immunohistochemical analysis included 34 patients (18 from NYU, 16 from Erasmus MC) with an average age of 41.6 years; 19 (56%) were male and 15 female. Mean (standard deviation [SD]) preoperative PRL concentration was 1796 (4772) ng/mL in 33 patients. Mean (SD) preoperative image transverse tumor size was 2.13 (1.61) cm in 15 patients for whom data were available. Mean (SD) postoperative PRL concentration was 108 (117) ng/mL in 17 patients for whom data were available.

Of the 34 patients, 31 (91%) used DA preoperatively at various doses. The most common indication for transsphenoidal resection of prolactinomas was DA resistance, which was present in 22 of 34 (64.7%) cases. Other indications for surgery included DA intolerance (*n* = 4) if experienced side effects preventing patients from continuing DA therapy, apoplexy (*n* = 2), optic chiasm compression (*n* = 5), cerebrospinal fluid leak (*n* = 3), suspected chordoma (*n* = 1), and patient preference (*n* = 2). Five cases had more than one of the above indications for surgery. In a significant number of cases, a dissociation between the effect of DA treatment on PRL level and tumor shrinkage was observed. In DA resistant patients from which both data on tumor shrinkage and PRL levels were available (*n* = 18), DA treatment resulted in normalization of PRL levels in 7 (39%), despite a lack of tumor shrinkage by more than 30% (supplementary Table 1).

### Immunohistochemical analysis of D2R, SST_2_ and SST_5_

**Fig. 1:**
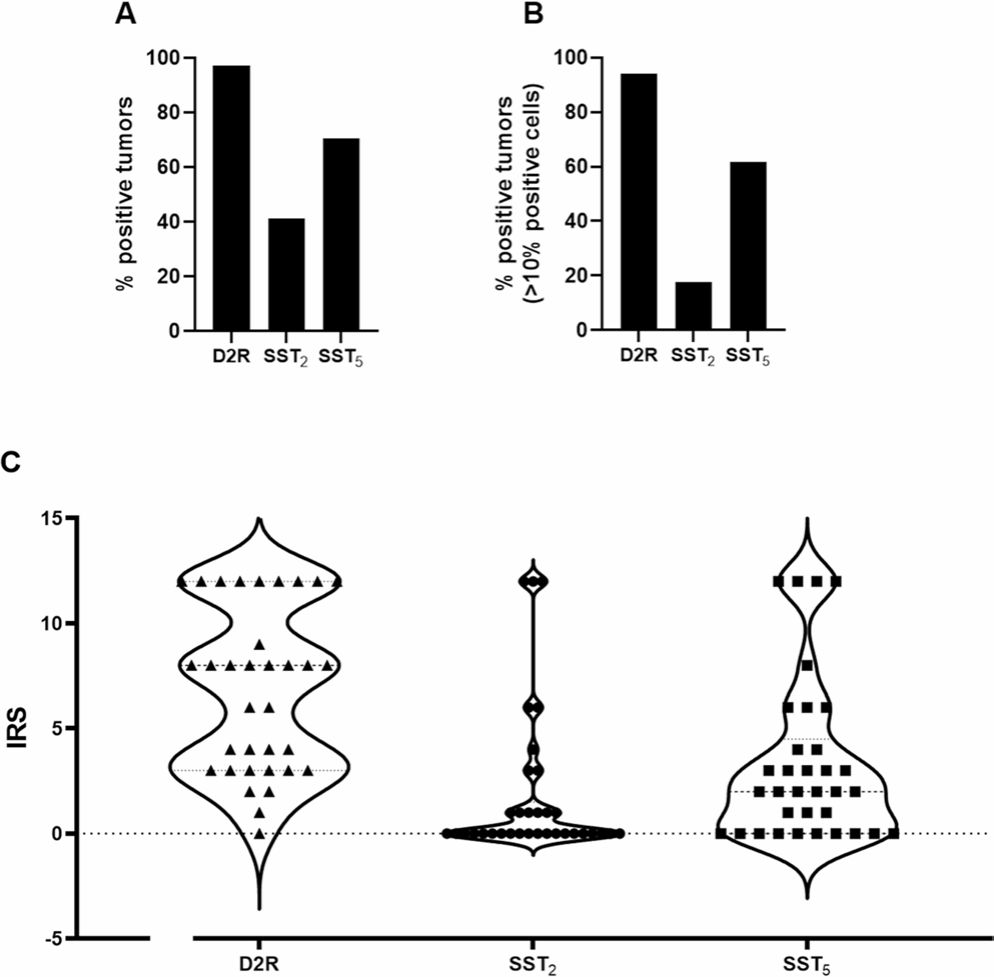
**A**: Percentage of tumors staining positive for D2R, SST_2_ and SST_5_; **B**: Percentage of tumors with > 10% D2R, SST_2_ and SST_5_; **C**: Violin plot showing IRS distribution for D2R, SST_2_ and SST_5_ amongst the cases

D2R was expressed in 97.1% (95% confidence interval [CI]: 80.8%, 99.2%) of cases (Fig. 1A). SST_5_ was expressed in 70.6% (95% CI: 43.6%, 77.9%) of prolactinomas (Fig. 1A) and expressed at a high level in a subset of these cases (IRS ≥ 6 in 8 of 34 cases). Only 41.2% (95% CI: 6.7%, 34.5%) of prolactinomas expressed SST_2_ (Fig. 1A). In the majority of these cases SST_2_ expression was very low (IRS ≤ 6 in 29 of 34 cases) and present in only a small percentage (< 10%) of tumor cells. Supplementary Fig. 1 shows the individual IRS scores in which tumors with < 10% positive cells are indicated by an asterisk. Since an expression of < 10% may be considered of no clinical relevance for response to DA, Fig. 1B also shows the percentage of tumors with > 10% D2R, SST_2_ or SST_5_ positive cells (94.1%, 17.6% and 61.8% positive tumors for D2R, SST_2_ and SST_5_, respectively).

**Fig. 2:**
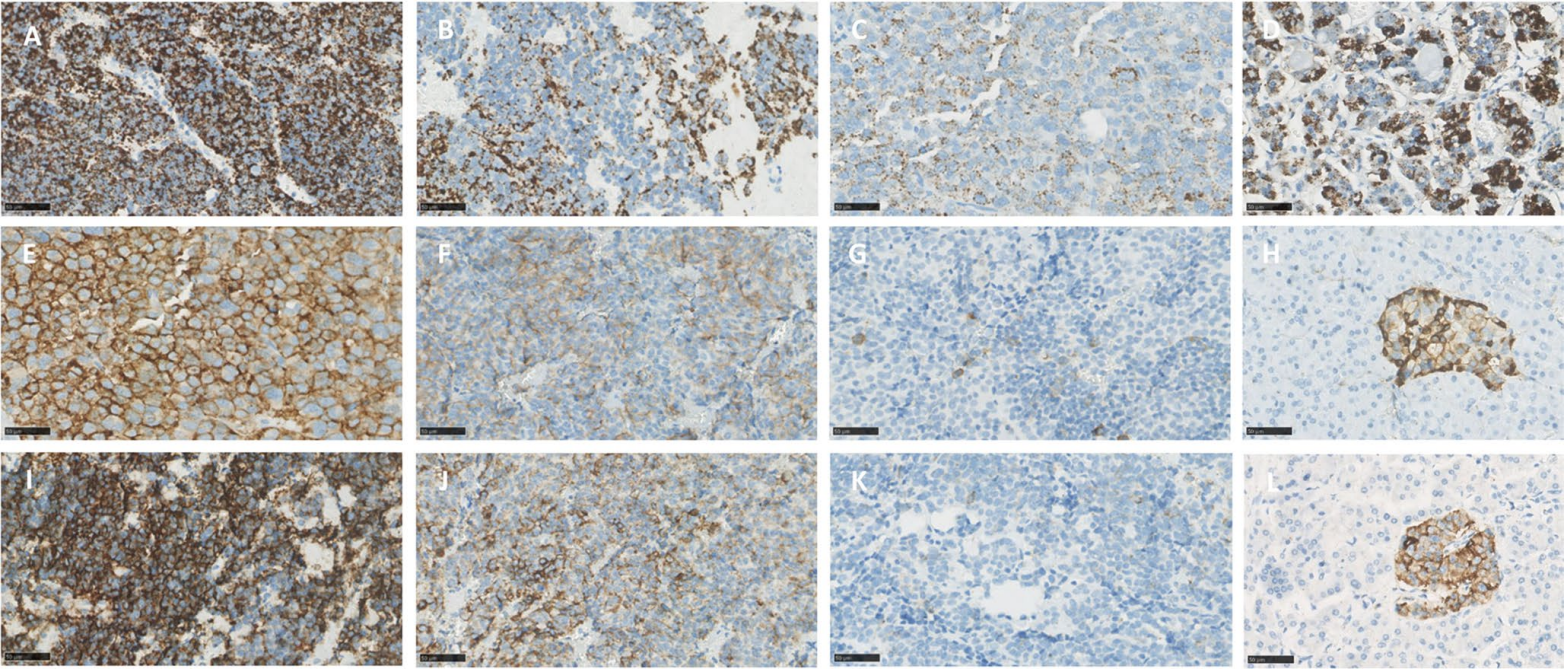
Representative photomicrographs of D2R, SST_2_ and SST_5_ immunohistochemical staining. Panels **A-D**: D2R, panels **E-H**: SST_2_, panels** I-L**: SST_5_. Panel **A**: case 2, IRS12; panel** B**: case 19, IRS6; panel **C**: case 10, IRS3; panel** D**: normal anterior pituitary gland; panel **E**: case 10, IRS12; panel** F**: case 31, IRS6; panel **G**: case 21, IRS1; panel **H**: normal pancreas; panel **I**: case 26, IRS 12; panel** J**: case 31, IRS6; panel** K**: case 2, IRS1; panel **L**: normal pancreas

Figure 1C shows a violin chart of the IRS scores for each receptor. For each receptor the minimum IRS was 0 and the maximum was 12. The median IRS score was 8.0 for D2R, 0.0 for SST_2_ and 2.0 for SST_5_. The 25th percentiles were 3.0 for D2R, 0.0 for SST_2_ and 0.0 for SST_5_ and the 75th percentiles were 12.0 for D2R, 1.5 for SST_2_ and 4.5 for SST_5_. Figure 2 shows representative photomicrographs of D2R, SST_2_ and SST_5_ immunohistochemical staining with high, intermediate, and low IRS scores. Localization of immunostaining for SST_2_ and SST_5_ was predominantly membranous, but displayed in some cases also some cytoplasmic staining. For D2R, staining pattern was granular with a localization on both the membrane and cytoplasm in all cases. Interestingly, we observed a moderate to strong expression of SST_2_ in small intratumoral vessels in most cases. Supplementary Fig. 2 shows a case with negative SST_2_ expression in tumor cells, but strongly SST_2_ positive small blood vessels.

The mean [SD] D2R IRS was lower in surgical specimens from patients with preoperative DA resistance compared to those without (5.27 [3.3] versus 9.67 [3.3], respectively; *p* < 0.001). Conversely, the mean [SD] D2R IRS was higher in specimens from patients with preoperative DA intolerance compared to those without (12.0 [0]) versus 6.32 [3.7], respectively; *p* = 0.014). Differences in mean IRSs for SST_5_ and SST_2_ between patients with preoperative DA resistance or intolerance and those without were not statistically significant. Furthermore, we found no differences in the mean SST_5_, SST_2_, or D2R IRSs with respect to the remaining clinico-radiological variables assessed (Table 1).

**Table 1:** Comparison of mean SST_5_, SST_2_, and D2R IRSs by presence of clinic-radiological features. Statistical significant values are highlighted in bold

Variable	SST_5_		SST_2_		D2R IRS	
	Mean (SD)	*P* value	Mean (SD)	*P* value	Mean (SD)	*P* value
CS Invasion*						
Yes (*n* = 9)	2.22 (2.9)	0.711	1.44 (4.0)	0.408	8.56 (3.9)	0.486
No (*n* = 8)	1.75 (2.1)		3.13 (4.2)		7.13 (4.4)	
Suprasellar Extension						
Yes (*n* = 27)	4.00 (4.1)	0.080	2.35 (4.0)	0.187	7.08 (3.9)	0.645
No (*n* = 7)	1.14 (1.2)		0.29 (0.5)		6.3 (4.6)	
Optic Chiasm Compression						
Yes (*n* = 5)	4.00 (4.7)	0.502	2.57 (4.3)	0.177	7.80 (4.6)	0.554
No (*n* = 29)	3.00 (3.2)		0.83 (1.6)		6.66 (3.9)	
Preoperative DA Therapy						
Yes (*n* = 31)	3.39 (4.0)	0.405	2.06 (3.7)	0.443	6.61 (3.8)	0.758
No (*n* = 2)	1.50 (2.1)		0 (0)		7.50 (6.4)	
Preoperative DA Resistance						
Yes (*n* = 22)	3.00 (3.5)	0.552	2.36 (3.6)	0.296	5.27 (3.3)	**< 0.001**
No (*n* = 12)	3.83 (4.4)		1.00 (3.5)		9.67 (3.3)	
Preoperative DA Intolerance						
Yes (*n* = 3)	1.33 (1.5)	0.361	0.00 (0.0)	0.348	12.00 (0.0)	**0.014**
No (*n* = 31)	3.48 (3.9)		2.06 (3.7)		6.32 (3.7)	
Apoplexy						
Yes (*n* = 2)	0.50 (0.7)	0.294	0.00 (0.0)	0.452	7.50 (6.4)	0.805
No (*n* = 32)	3.47 (3.9)		2.00 (3.6)		6.78 (3.9)	

*Data available for 18 of 34 patients

Correlation analysis showed no statistically significant relationship between tumor volume or preoperative PRL level and SST_5_, SST_2_, or D2R IRSs. Furthermore, our analysis did not reveal a significant relationship between SST_5_, SST_2_, and D2R IRSs (Supplementary Table 2).

### Effects of octreotide, Pasireotide and Cabergoline on PRL secretion by primary cultures of prolactinoma cells

**Fig. 3:**
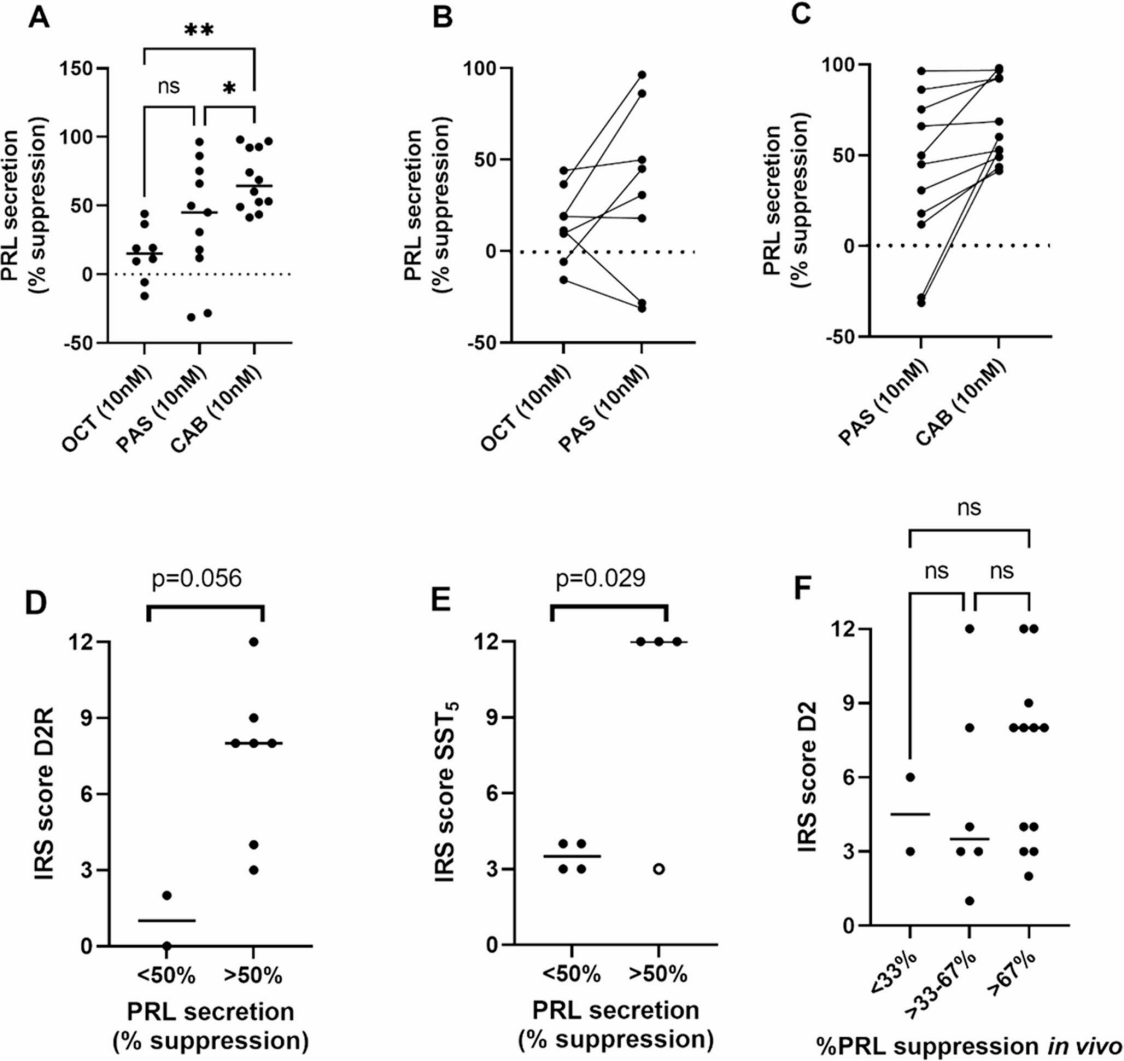
(**A)** The effect of treatment with octreotide (OCT; 10nM; *n* = 8), Pasireotide (PAS; 10nM; *n* = 11) and Cabergoline (CAB; 10nM; n=12) on PRL secretion by primary cultured human prolactinoma cells. In one case, bromocriptine (10nM) was used instead of cabergoline. Values represent the % inhibition of prolactin secretion. Horizontal lines represent the median value. *p < 0.05; **p < 0.01; (**B**) Paired analysis of the effects of OCT (10nM) and PAS (10nM) in vitro, (**C**) Paired analysis of the effects of PAS (10nM) and CAB (10nM) in vitro, (**D**) relation D2R IRS score and % inhibition of PRL secretion by CAB (10nM) (categorized as < 50% or > 50% inhibition compared to untreated control), **(E)** relation SST_5_ IRS score and % inhibition of PRL secretion by PAS (10nM) in vitro (categorized as < 50% or > 50% inhibition compared to untreated control). Open circle: statistical outlier, **(F)** relation D2R IRS score and % inhibition of PRL secretion by DA treatment in vivo (categorized as < 33, >33–67% and > 67% inhibition compared to pre-treatment level)

Figure 3 summarizes the results of the in vitro studies comparing the effects of OCT, PAS and CAB on PRL secretion by cultured primary human prolactinoma cells. OCT was the least potent compound (Fig. 3A). Overall, CAB was the most potent drug in vitro (median PRL suppression by OCT 15.1%, compared to 45.0 and 64.5% for PAS and CAB, respectively). In a subgroup of prolactinoma cultures PAS inhibited PRL secretion with comparable potency to cabergoline (Fig. 3A). When the efficacy of the three drugs was compared simultaneously in the same cultures (*n* = 8) a similar rank order of potency (CAB > PAS > OCT) was found (supplementary Fig. 3). We did not observe a statistically significant difference in the inhibition of PRL secretion by OCT, PAS and CAB between adenomas from patients with DA resistance and adenomas resected for other indications (supplementary Fig. 4).

In 5 of 8 cultures where OCT and PAS were directly compared, PAS was more potent in suppressing PRL secretion (median inhibition 19.2% by OCT and 49.9% by PAS in the 5 cultures; Fig. 3B). In 9 of 11 cultures, CAB was more potent than PAS in suppressing PRL secretion (median inhibition 53.1% by CAB and 30.7% by PAS in the 9 cultures; Fig. 3C).

D2R IRS score was highest in cultures showing > 50% inhibition of PRL secretion by CAB (median IRS 1.0 for < 50% inhibition and 8.0 for > 50% inhibition; Fig. 3D), while cultures that showed the highest response to PAS had the highest SST_5_ IRS score (median IRS 3.5 for < 50% inhibition and 12.0 for > 50% inhibition; Fig. 3E). Moreover, there was a good agreement between the percentage of suppression of PRL secretion by DA treatment in vitro and in vivo. Except for one case, the inhibition of PRL secretion in vivo (median inhibition 99.4%) was slightly higher compared to the effect of DA in vitro (median inhibition 68.7%; supplementary Fig. 5). Figure 3F shows the D2R IRS scores and the response of PRL levels after in vivo DA treatment, categorized in tertiles. Although there was a trend towards higher D2 expression in patients showing the strongest lowering of PRL levels in vivo, the differences did not reach statistical significance (median D2R IRS 4.5, 3.5 and 8.0 for PRL suppression < 33%, > 33–67%, and > 67%, respectively).

## Discussion

Approximately 10% of prolactinomas are resistant to DA therapy, and a substantial number of patients are intolerant to DAs owing to their adverse effects [11, 14].

DA resistance is not well understood, but multiple possible mechanisms have been hypothesized. Biochemical studies suggest that it may be due to decreased dopamine receptor expression or alterations in intracellular signaling that modulate dopamine receptor expression and signaling downstream from dopamine receptors [15]. Studies have shown a decreased density of D2Rs in cells of DA-resistant prolactinomas compared with cells of DA-sensitive prolactinomas [16]. Expression of D2R mRNA is reduced 4-fold in DA-resistant tumors compared with DA-sensitive tumors; D2R gene transcription is in turn reduced, which ultimately reduces the number of D2Rs [3, 15]. Another proposed mechanism involves an imbalance between the two D2R isoforms, the short isoform D2S and the long isoform D2L.There is equivocal data regarding which D2R isoform contributes more to DA resistance. Szmygin et al. [3] described that dopamine is thought to exert its inhibitory effects on lactotroph cells primarily via the D2S isoform, and DA-resistant prolactinomas express lower levels of D2S isoform mRNA and higher levels of D2L isoform mRNA than do DA-sensitive tumors. This study also described that estrogens may play a role in the development of DA-resistant prolactinomas by increasing expression of the D2L isoform [3, 17]. However, Shimazu et al. showed that resistance may be related to lower levels of D2L rather than changes to D2S [18]. Pivonello et al. in a recent review also indicated that lower expression of D2S and D2S/D2L ratio was associated with DA resistance in prolactinomas [7]. Other factors may contribute to DA resistance as well, including overexpression of growth factors VEGF and EGF; prolactin receptor variants; a variant in the gene Ncol- T+, which is associated with decreased D2R production; and alterations in receptors that can indirectly affect D2R signaling such as NGFR [1, 3, 7, 15, 19], although none have been conclusively implicated.

While dopamine receptor expression predominates in prolactinomas, these tumors also express SST_1–5_ to varying degrees [15]. Jaquet et al. found that SST_5_ mRNA expression predominates in prolactinomas, followed by SST_1_ and SST_2_ mRNA expression [9]. In the present study, a similar order of expression of SST_5_ > SST_2_ was observed at the protein level. Whereas the present study found that a relative high proportion of prolactinomas are positive for SST_5_, a prior study by Raverot et al. found significant SST_5_ expression in only 3 of 21 macroprolactinomas studied (14%) [20]. Of note, in our study only 5 out of 34 prolactinomas (15%) displayed a high SST_5_ expression (IRS ≥ 8). These results suggest that SST_5_ expression is highly variable among prolactinomas and SST expression analysis might be useful to guide therapy for DA-resistant tumors after pituitary surgery [11]. This variability may be influenced by multiple factors, including intrinsic genetic differences, epigenetic modifications, and prior treatment effects which may influence SST_5_ expression [7, 8].

While a number of studies have evaluated the expression of SST and D2 receptors among prolactinomas, our study is unique in that it evaluated the expression of SST_2_, SST_5_ and D2R within a single cohort. The study conducted by Thodou et al. in 2006 evaluating the expression of SST receptors in pituitary adenomas (growth hormone and prolactin secreting), found that among lactotroph adenomas, the predominant receptor was SST_5_, followed by SST_1_. However, in this study the concomitant expression of D2 receptors on these specimens was not evaluated [21]. In our series of 34 patients with prolactinomas, which is, to the best of our knowledge, the largest prolactinoma series for SST and D2R receptor immunohistochemical staining, 22 (64.7%) were DA-resistant and 4 (11.8%) intolerant to DA therapy. It has been hypothesized that prolactinomas resistant to DA have a reduced number of D2Rs. Consistent with this hypothesis, Fusco et al. found that although D2R mRNA was the predominant receptor among all tumor tissues studied, mean D2R mRNA levels were significantly lower in DA-resistant tumors than in DA-sensitive tumors [8]. Of note, mRNA levels of D2R, SST_2_ and SST_5_ were quantified in this study, rather than protein expression using immunohistochemical staining. Results from the present study showed that the mean D2R IRS was significantly lower among patients with preoperative DA resistance than among those without DA resistance. Dopamine is thought to exert its inhibitory effects on lactotroph cells primarily via the D2S isoform, and DA-resistant prolactinomas express lower mRNA levels of the D2S isoform and higher levels of the D2L isoform than do DA-sensitive tumors [1, 3]. The antibody we used for D2R staining identifies the presence of the D2R receptor as such, but does not distinguish between the two different isoforms that D2R exists in, namely D2 short (D2S) and D2 long (D2L). This may explain the high D2R receptor positivity amongst samples with DA resistance. SST_5_ specifically regulates PRL secretion from human prolactinoma cells in vitro [8, 9, 22]. Although expression of SST_1_ mRNA in human prolactinomas is equivalent to or greater than that of SST_5_ mRNA, in vitro studies found no correlation between SST_2_ and SST_1_ mRNA expression and inhibition of PRL secretion [8, 9]. Shimon et al. assessed the involvement of SST_2_ and SST_5_ in somatotroph and lactotroph tumors by studying in vitro growth hormone and PRL secretion from these tumors using SRLs with selective affinity for SST_2_ or for SST_5_. Among six prolactinomas, SRLs selective for SST_5_ significantly suppressed in vitro secretion of PRL in four cases, two of which were DA-resistant [22]. SRLs selective for SST_2_ did not suppress PRL release from any of the six tumors. These results demonstrate the potential for SRLs selective for SST_5_ to suppress PRL secretion from prolactinomas, including DA-resistant tumors [22]. Jaquet et al. found that suppression of PRL secretion by BIM23268, a SRL selective for SST_5_, was correlated with quantitative SST_5_ receptor expression among 10 patients with prolactinomas, 3 of which were DA-resistant. They observed a dose-dependent decrease in PRL secretion with a SRL selective for SST_5_ in vitro, which supports the concept that SST_5_ is the primary SST subtype controlling PRL secretion [9].

Fusco et al. studied 10 patients with prolactinomas and found that BIM23206, a SRL selective for SST_5,_ could inhibit PRL in a dose-dependent manner similar to cabergoline in DA-sensitive prolactinomas but only partially suppressed PRL secretion in DA-resistant prolactinomas. The combination of BIM23206 and cabergoline induced a dose-dependent inhibition of PRL secretion, like that produced by cabergoline alone, in DA-resistant tumors [8].

Although SST_5_-selective SRLs can inhibit PRL release in human prolactinomas, the in vitro studies conducted to date show that they have a weaker effect on PRL inhibition than do DAs [8, 9]. Similarly, the results of our study showed a rank order of potency of inhibition of PRL secretion in vitro CAB > PAS > > OCT.

Lasolle et al. and Coopmans et al. demonstrated the efficacy of pasireotide to normalize PRL secretion and induce tumor shrinkage in patients with DA-resistant prolactinomas [11, 23]. In both case reports, SST_2_ and SST_5_ were analyzed by immunohistochemistry and IRSs were calculated, similar to the present study. IRSs for SST_5_ were moderate or high in both tumor specimens, suggesting that SST_5_ expression might be useful to predict tumor response to PAS [11, 23]. In both the above studies, it was shown that PAS therapy might induce cystic degeneration, tumor necrosis, or both, which indicates the potential anti-tumor effects PAS may separate from normalization of prolactin levels [11, 23]. PAS has been shown to induce tumor shrinkage in corticotroph and somatotroph tumors as well [24]. Interestingly, in our study we also observed significant expression of SST_2_ in small intratumoral vessels, even in prolactinomas in which the tumor cells were SST_2_ negative. A previous study reported the presence of SST_2_ on intratumoral vessels in LH/FSH producing pituitary tumors, but not in GH-producing tumors [25]. Therefore, in addition to LH/FSH producing pituitary tumors, the current study shows that also prolactinomas display SST_2_ expression on intratumoral blood vessels. Whether this observation has any clinical significance is currently not known.

Our in vitro data comparing the effects of OCT (high SST_2_ affinity) and PAS (high SST_2_ and SST_5_ affinity) are concordant with the above observations. We found that OCT was not effective in suppressing PRL secretion in vitro, whereas PAS potently suppressed PRL secretion in a subset of prolactinoma cultures. The effect of PAS was highest in the prolactinomas with high SST_5_ protein expression, which confirms the role of SST_5_ in inhibiting PRL secretion. Consistent with results from other studies, DA were more potent compared to PAS in suppressing PRL secretion. On the other hand, in a subset of prolactinomas CAB and PAS were equally effective in lowering prolactin secretion. This was an interesting finding which may define the potential role of PAS in DA resistance and discrepant findings of good biochemical control and poor tumoral control. These discrepant findings may also provide an explanation for our observation that there were no statistically significant differences between the effects of OCT, PAS or CAB on PRL secretion by prolactinomas from DA resistant patients and from patients operated for other reasons.

Limitations of this study include the relatively small sample size and the retrospective nature of this proof-of-concept study. Future prospective studies with larger samples are needed to validate these findings. Another limitation is the heterogeneous nature of the sample, which included patients with DA resistance, DA intolerance, and other indications for surgery; this variability may introduce bias and affect the generalizability of the findings. Given some limitations in data available regarding pre-operative CAB cumulative dose and duration, we were not able to correlate these to SST_2_ or SST_5_ expression, which may have been helpful in determining whether prolonged DA therapy before surgery influences SST expression and responsiveness to SRLs. In addition, the in vitro experiments evaluated PRL secretion, but did not include assessment of cell growth markers, which may have provided data on the potential anti-tumor effects of pasireotide. A strength of our study is that we evaluated concomitant SST_2_, SST_5_ and D2R expression at the protein level and established the relationship between SST_2_, SST_5_ and D2R expression and effects of SST_2_, SST_5_ and D2R selective ligands. To the best of our knowledge, our study is the largest in vitro study evaluating the effects of OCT, PAS and CAB in vitro in prolactinoma cultures.

In conclusion, targeting SST_5_ with PAS may be a potential treatment modality for further clinical investigation in the treatment of a subset of DA resistant or intolerant prolactinomas.

## Supplementary Information

Below is the link to the electronic supplementary material.

Supplementary Material 1 (PDF 415 KB)

## Data Availability

No datasets were generated or analysed during the current study.
