# Supplementary material for: Somatostatin 5 receptor expression in prolactinomas: Is there a role for Pasireotide in the management of prolactinomas?
Source: Pituitary. 2025 Nov 3;28(6):123. doi: 10.1007/s11102-025-01580-7 (PMC12583388; doi:10.1007/s11102-025-01580-7)
Supplement: Supplementary file 1 — Supplementary Material 1 (PDF 415 KB) [file 11102_2025_1580_MOESM1_ESM.pdf]

**Supplementary table 1: Demographic and individual data.**

| Case | Age at Surgery | Sex (M/F) | Indication for surgery | Normalization of PRL by DA treatment (Y/N) | ≥30% tumor shrinkage by DA treatment (Y/N) | Percent PRL suppression by DA treatment** | IRS $SST_2$ | IRS $SST_5$ | IRS D2R | In vitro effect OCT (10nM)** | In vitro effect Pas (10nM)** | In vitro effect Cab (10nM)** |
|---|---|---|---|---|---|---|---|---|---|---|---|---|
| 1 | 18 | F | Dopamine agonist resistance | N | n.a. | 90.2 | 6 | 0 | 8 | n.a. | n.a. | n.a. |
| 2 | 49 | M | Dopamine agonist intolerance; patient preference | | | n.a. | 0 | 1* | 12 | n.a. | n.a. | n.a. |
| 3 | 53 | F | Dopamine agonist resistance; optic chiasm compression | N | N | **-71.7** | 0 | 0 | 3 | n.a. | n.a. | n.a. |
| 4 | 32 | M | Prolactinoma with apoplexy; optic chiasm compression | | | n.a. | 0 | 0 | 3 | n.a. | n.a. | n.a. |
| 5 | 45 | M | Prolactinoma with apoplexy; optic chiasm compression | | | n.a. | 0 | 1* | 12 | n.a. | n.a. | n.a. |
| 6 | 29 | F | Dopamine agonist resistance | Y | N# | n.a. | 3* | 2 | 4 | n.a. | n.a. | n.a. |
| 7 | 32 | M | Dopamine agonist resistance | N | N | 82.9 | 0 | 6 | 2 | n.a. | n.a. | n.a. |
| 8 | 54 | M | Suspected chordoma | | | n.a. | 0 | 8 | 12 | n.a. | n.a. | n.a. |
| 9 | 39 | F | Dopamine agonist intolerance | | | n.a. | 0 | 3 | 12 | n.a. | n.a. | n.a. |
| 10 | 54 | M | Dopamine agonist resistance | N | N | 51.3 | 12 | 2 | 3 | n.a. | n.a. | n.a. |
| 11 | 19 | F | Dopamine agonist resistance | N | n.a. | 60.1 | 1* | 0 | 8 | n.a. | n.a. | n.a. |
| 12 | 39 | F | Dopamine agonist resistance; patient preference | N | N | 52.2 | 3* | 6 | 12 | n.a. | n.a. | n.a. |
| 13 | 31 | F | Cerebrospinal fluid leak | | | n.a. | 12 | 2 | 8 | n.a. | n.a. | n.a. |
| 14 | 40 | F | Dopamine agonist resistance | N | N | 65.7 | 1* | 0 | 3 | n.a. | n.a. | n.a. |
| 15 | 51 | M | Optic chiasm compression | | | n.a. | 0 | 3 | 12 | n.a. | n.a. | n.a. |
| 16 | 46 | M | Cerebrospinal fluid leak | | | n.a. | 0 | 0 | 8 | n.a. | n.a. | n.a. |
| 17 | 40 | F | Dopamine agonist resistance | N | n.a. | 41.0 | 1* | 0 | 4 | n.a. | n.a. | n.a. |
| 18 | 21 | F | Dopamine agonist intolerance | | | n.a. | 0 | 0 | 12 | n.a. | n.a. | n.a. |
| 19 | 33 | M | Dopamine agonist resistance | n.a. | N | n.a. | 1* | 12 | 6 | n.a. | n.a. | n.a. |
| 20 | 37 | F | Dopamine agonist resistance | Y | N | 99.6 | 0 | 0 | 3 | n.a. | n.a. | n.a. |
| 21 | 34 | M | Dopamine agonist resistance | n.a. | N | n.a. | 1* | 2 | 8 | n.a. | n.a. | 74.3 |
| 22 | 32 | F | Cerebrospinal fluid leak | Y | Y | 99.8 | 0 | 12 | 4 | n.a. | 66 | 68.7 |
| 23 | 55 | M | Dopamine agonist resistance | N | N | n.a. | 0 | 3 | 2 | 9.5 | 30.7 | 49.1 |
| 24 | 48 | M | Optic chiasm compression | N | Y (later apoplexia) | 94.0 | 0 | 12 | 9 | n.a. | 75.3 | 92.7 |
| 25 | 15 | F | Dopamine agonist resistance | N | N | 57.4 | 12 | 3 | 1* | 18.9 | 17.9 | 41.4 |
| 26 | 42 | M | Dopamine agonist resistance | Y | N | 100.0 | 0 | 12 | 8 | 36.5 | 96.4 | 96.9 |
| 27 | 43 | M | Dopamine agonist resistance | Y | Y (regrowth after 1 yr treatment) | 100.0 | 0 | 3 | 3 | 19.2 | 86.2 | 92.3 |
| 28 | 44 | M | Dopamine agonist resistance | N | Y (regrowth after 8 months treatment) | 99.4 | 0 | 4 | 8 | **-15.7** | **-31.3** | 53.1 |
| 29 | 33 | M | Dopamine agonist intolerance | N | N | 69.9 | 0 | 4 | 12 | 11.3 | **-28.3** | 60.2 |
| 30 | 85 | F | Dopamine agonist resistance | N | Y (regrowth after 2 yr treatment) | 68.2 | 4 | 0 | 8 | n.a. | n.a. | n.a. |
| 31 | 48 | M | Dopamine agonist resistance | Y | N | 100.0 | 6 | 6 | 12 | n.a. | n.a. | n.a. |
| 32 | 50 | M | Dopamine agonist resistance | Y | N | 98.3 | 1* | 1* | 4 | n.a. | n.a. | n.a. |
| 33 | 69 | F | Dopamine agonist resistance | N | Y (regrowth after several years) | n.a. | 0 | 2 | 0 | n.a. | n.a. | n.a. |
| 34 | 36 | M | Dopamine agonist resistance | N | Y (initially full regression with cabergoline, later regrowth) | **-227.0** | 0 | 2 | 6 | n.a. | n.a. | n.a. |
| 35 | 42 | M | Dopamine agonist resistance | Y | N | n.a | n.a. | n.a. | n.a. | n.a. | 11.9 | 43.5 |
| 36 | 36 | F | Dopamine agonist resistance | Y | N | n.a | n.a. | n.a. | n.a. | 44.1 | 49.9 | 98 |
| 37 | 37 | F | Dopamine agonist intolerance | N | N | **-318.0** | n.a. | n.a. | n.a. | -5.8 | 45 | 52.7 |

# Suspect radiologic nonresponse on DA therapy. Pre-op MRI showed >1cm macroadenoma, but MRI with measurements on initial pre-DA MRI not available.

** minus value means increase

* cases with <10% positive tumor cells

n.a. not available

Columns G, K and L: values in bold represent an increase

**Supplementary table 2**

Results of correlation analysis between $SST_5$, $SST_2$, and D2R IRSs and clinic-radiological features. Abbreviations: PCC, Pearson correlation coefficient; NA, not applicable.

| | **SSTR5 IRS** | | **SSTR2a IRS** | | **D2R IRS** | |
|---|---|---|---|---|---|---|
| Variable | PCC | p-value | PCC | p-value | PCC | p-value |
| Age at surgery | -0.01 | 0.936 | -0.16 | 0.358 | -0.01 | 0.967 |
| Tumor volume* | 0.07 | 0.828 | -0.31 | 0.353 | 0.25 | 0.464 |
| Preoperative PRL | -0.14 | 0.428 | -0.16 | 0.367 | -0.22 | 0.212 |
| SSTR5 IRS | NA | NA | -0.12 | 0.519 | 0.14 | 0.439 |
| SSTR2a IRS | NA | NA | NA | NA | -0.13 | 0.467 |

*Data of tumor volume available for 18 of 34 patients.

**Supplementary figure 1**

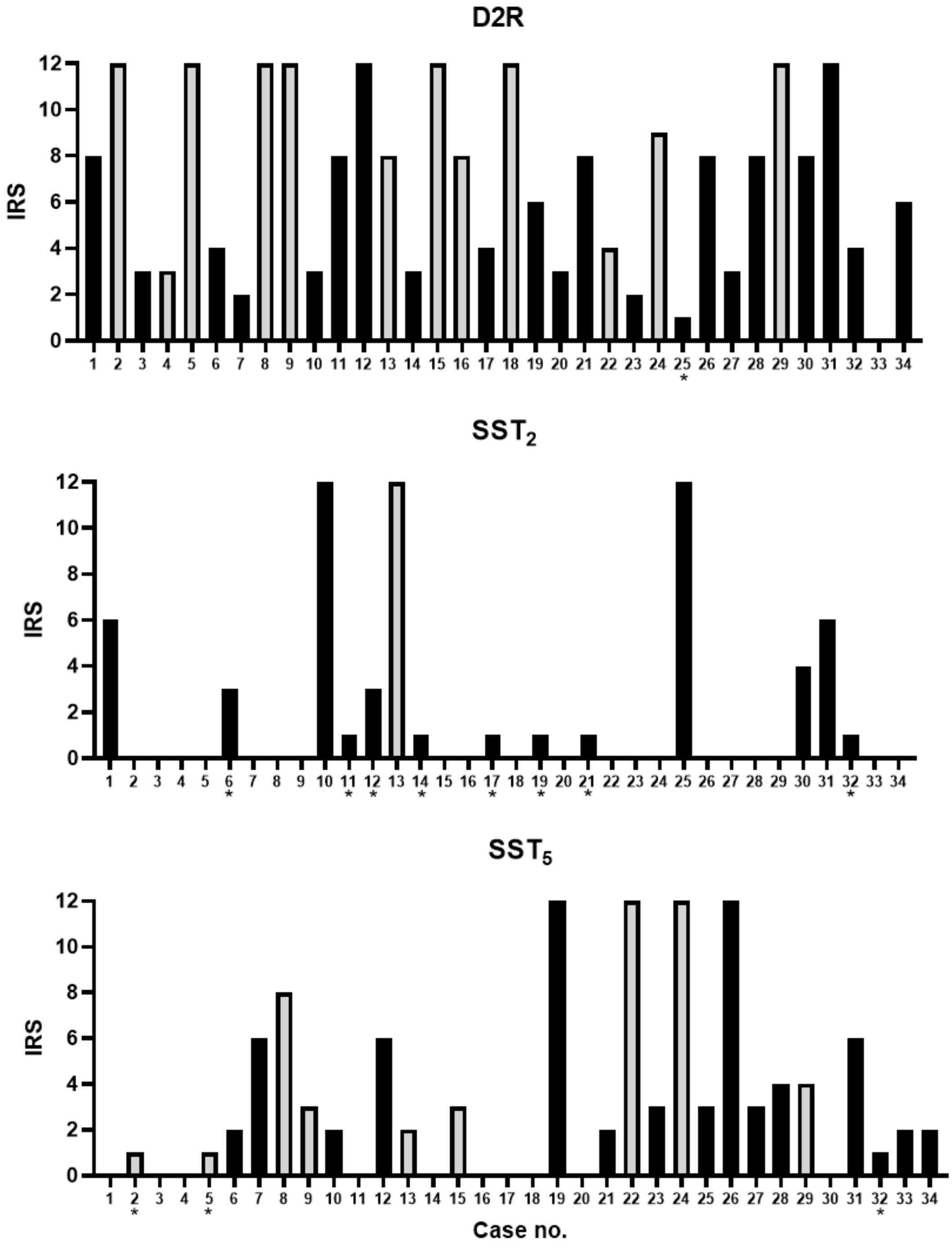


Individual IRS scores for D2R (upper panel), $SST_2$ IRS (middle panel) and $SST_5$ (lower panel). Cases of tumors with <10% positive cells are marked with an asterisk. Black filled bars: tumors of resistant patients; gray filled bars: tumors of patients operated for other reasons.

**Supplementary figure 2**

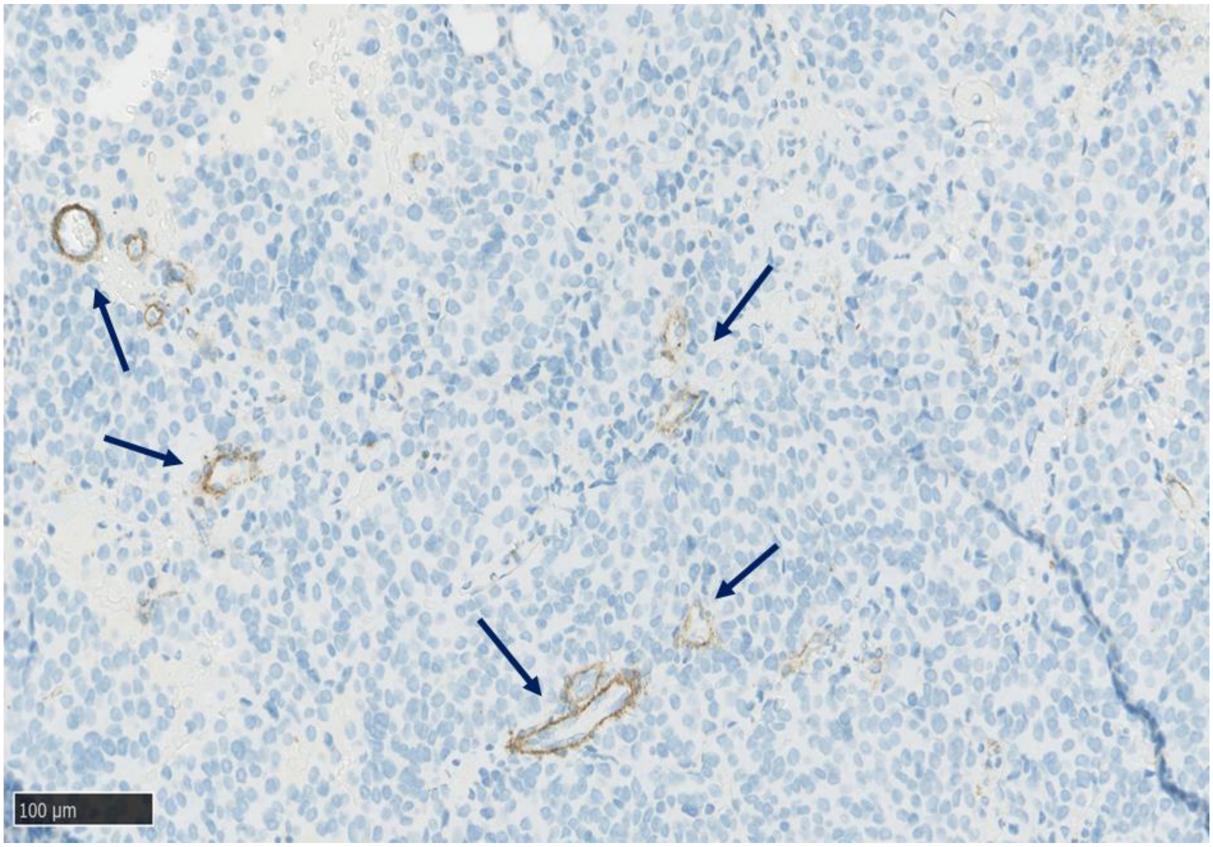


Representative photomicrograph of a prolactinoma with $SST_2$ negative tumor cells, but with $SST_2$ expression in small vessels (arrows).

**Supplementary figure 3**

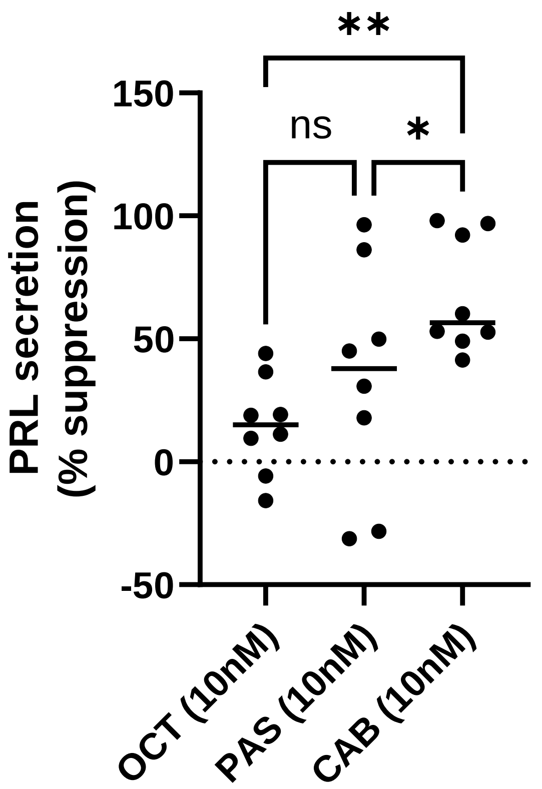


The effect of treatment with OCT, PAS and CAB (all at 10nM concentration) on PRL secretion by primary cultured human prolactinoma cells. All drugs were tested simultaneously in the same cultures (n=8). Values represent the % suppression of prolactin secretion compared to untreated control cells. Horizontal lines represent the median value. *p<0.05; **p<0.01.

**Supplementary figure 4**

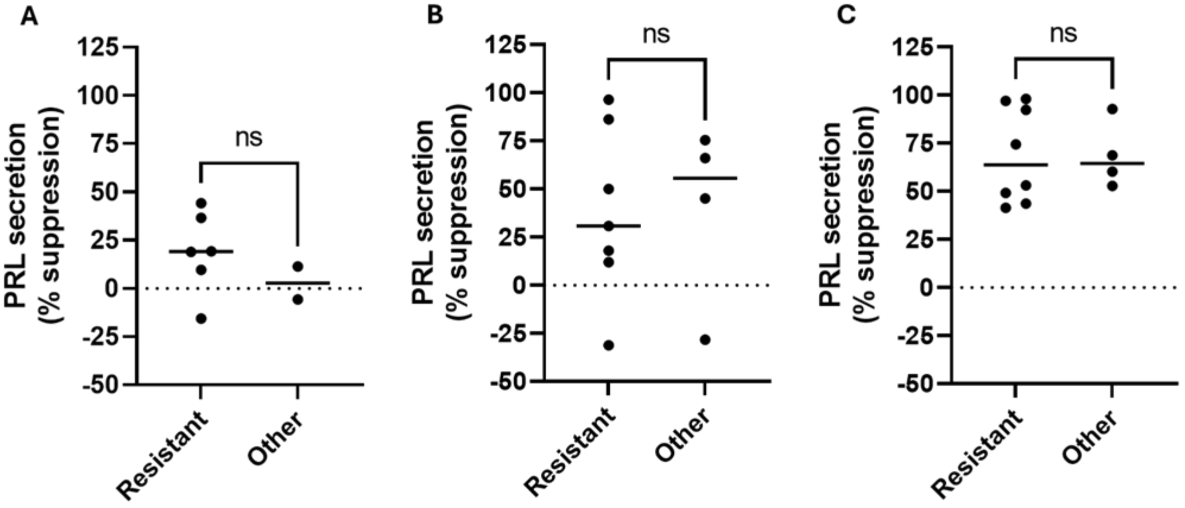


Comparison between the effect of 10nM OCT (A), 10nM SOM (B) and 10nM CAB (C) treatment *in vitro* PRL secretion by cultures of prolactinomas of DA-resistant patients (Resistant) and of patients that were DA-intolerant or operated for other reasons (Other). A negative value means increase in PRL level *in vitro*.

**Supplementary figure 5**

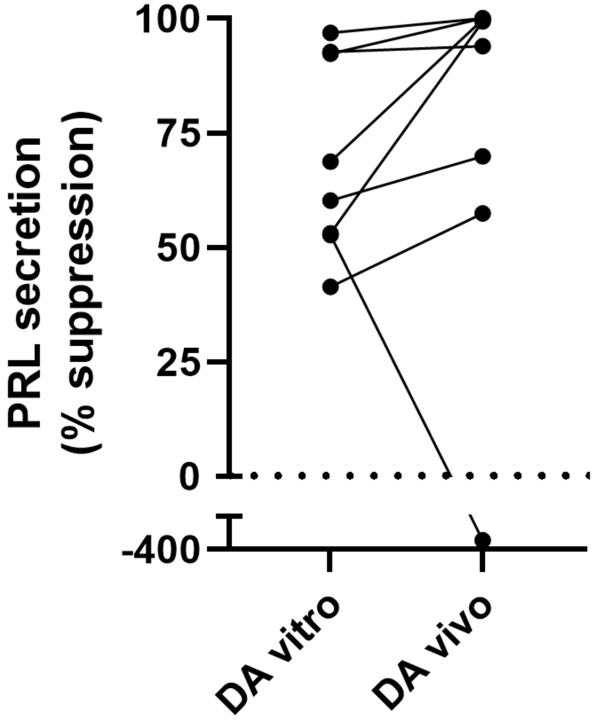


Comparison between the effect of DA treatment in vitro and in vivo (n=8; ns). A negative value means increase in PRL level *in vivo*.
